# Neuropathological Lesions and Cognitive Abilities in Black and White Older Adults in Brazil

**DOI:** 10.1001/jamanetworkopen.2024.23377

**Published:** 2024-07-25

**Authors:** Claudia K. Suemoto, Renata E. P. Leite, Vitor R. Paes, Roberta Rodriguez, Alberto F. O. Justo, Michel S. Naslavsky, Mayana Zatz, Carlos A. Pasqualucci, Ricardo Nitrini, Eduardo Ferriolli, Wilson Jacob-Filho, Lea T. Grinberg

**Affiliations:** 1 Division of Geriatrics, University of Sao Paulo Medical School, Sao Paulo, Sao Paulo, Brazil; 2 Department of Pathology, University of Sao Paulo Medical School, Sao Paulo, Sao Paulo, Brazil; 3 Department of Neurology University of Sao Paulo Medical School, Sao Paulo, Sao Paulo, Brazil; 4 Human Genome and Stem Cell Center, Biosciences Institute, University of Sao Paulo, Sao Paulo, Sao Paulo, Brazil; 5 Memory and Aging Center, University of San Francisco, San Francisco, California; 6 Global Brain Health Institute, University of San Francisco, San Francisco, California

## Abstract

**Importance:**

Race differences in dementia prevalence and incidence have previously been reported, with higher dementia burden in Black decedents. However, previous neuropathological studies were conducted mostly in convenience samples with White participants; conducting clinicopathological studies across populations is crucial for understanding the underlying dementia causes in individuals from different racial backgrounds.

**Objective:**

To compare the frequencies of neuropathological lesions and cognitive abilities between Black and White Brazilian adults in an autopsy study.

**Design, Setting, and Participants:**

This cross-sectional study used samples from the Biobank for Aging Studies, a population-based autopsy study conducted in Sao Paulo, Brazil. Participants were older adults whose family members consented to the brain donations; Asian participants and those with missing data were excluded. Samples were collected from 2004 to 2023. Neuropathologists were masked to cognitive outcomes.

**Exposure:**

Race as reported by the deceased’s family member.

**Main Outcomes and Measures:**

The frequencies of neurodegenerative and cerebrovascular lesions were evaluated in 13 selected cerebral areas. Cognitive and functional abilities were examined with the Clinical Dementia Rating Scale.

**Results:**

The mean (SD) age of the 1815 participants was 74.0 (12.5) years, 903 (50%) were women, 617 (34%) were Black, and 637 (35%) had cognitive impairment. Small vessel disease (SVD) and siderocalcinosis were more frequent in Black compared with White participants (SVD: odds ratio [OR], 1.74; 95% CI, 1.29-2.35; *P* < .001; siderocalcinosis: OR, 1.70; 95% CI, 1.23-2.34; *P* = .001), while neuritic plaques were more frequent in White compared with Black participants (OR, 0.61; 95% CI, 0.44-0.83; *P* = .002). Likewise, Alzheimer disease neuropathological diagnosis was more frequent in White participants than Black participants (198 [39%] vs 77 [33%]), while vascular dementia was more common among Black participants than White participants (76 [32%] vs 121 [24%]). Race was not associated with cognitive abilities, nor did it modify the association between neuropathology and cognition.

**Conclusions and Relevance:**

In this cross-sectional study of Brazilian older adults, Alzheimer disease pathology was more frequent in White participants while vascular pathology was more frequent in Black participants. Further neuropathological studies in diverse samples are needed to understand race disparities in dementia burden.

## Key Points

**Question:**

Do neuropathology and cognitive abilities differ by race?

**Findings:**

In a cross-sectional autopsy study with 1815 Black and White older adults in Brazil, neuritic plaques were less frequent in Black participants than White participants, while small vessel disease and siderocalcinosis were more frequent. Alzheimer disease was more common among White than Black participants, while vascular dementia was more common among Black than White participants; however, cognitive abilities were similar between Black and White participants.

**Meaning:**

This study found differences in frequency of specific neuropathologies by race, but no difference in cognitive impairment.

## Teaser

This cross-sectional study evaluates differences in the frequency of neuropathological lesions and cognitive outcomes among Black and White older adults in Brazil.

## Introduction

Emerging epidemiological evidence suggests that there are racial disparities in the prevalence and incidence of dementia with higher rates in Black decedents.^1,2^ Neuropathological assessment is key to determining the cause of dementia because it can evaluate the presence and severity of neuropathologies.^3^ However, most studies were performed on White populations.^4^ The few studies conducted on decedents from other racial and ethnic groups have shown conflicting results, either suggesting that Black decedents have a higher burden of Alzheimer disease (AD) pathology and cerebrovascular disease and a higher frequency of multiple pathologies than non-Hispanic White participants^5,6^ or that Black decedents have less AD burden.^7^

As most studies have been performed in the United States and Europe,^5,6^ it is important to explore this association in other populations to determine the generalizability of these findings. Moreover, studies included convenience samples, most from research centers, which receive brain donations mostly from people with advanced dementia with a very modest number of Black decedents.^5,6^ Population-based autopsy studies are key to overcoming these limitations. Therefore, our study aimed to investigate the association of reported race with the frequency of age-related neuropathology and dementia in a large and racially and ethnically diverse sample from Brazil, which provides a unique opportunity to investigate race disparities in neuropathology in a population with different genetic and environmental backgrounds.

## Methods

### Participants

This study used data from the Biobank for Aging Studies (BAS) at the University of Sao Paulo Medical School, which has a collection of 1990 brains donated between 2004 and 2023. A detailed description of the BAS procedures can be found elsewhere.^8^ The deceased’s next of kin (NOK) signed an informed consent for brain donation, and the BAS was approved by local and national research committees. This study followed the Strengthening the Reporting of Observational Studies in Epidemiology (STROBE) reporting guidelines for cross-sectional studies.

The BAS inclusion criteria are being aged 18 years or older by the time of death and having a knowledgeable NOK who had at least weekly contact with the deceased in the 6 months before death. We excluded individuals with a postmortem interval greater than 24 hours and those whose brain tissue was not suitable for neuropathological analyses (eg, cerebrospinal fluid pH <6.5).

### Clinical Assessment

Sociodemographic variables included age, sex, education, and race. The NOK reported the number of years of education and race. The Brazilian Institute of Geography and Statistics (IBGE), which conducts the Brazilian census, classifies race into 5 categories: White, Black, *Pardo* (admixture of Black and White race), Indigenous, and Asian.^9^ The interviewer asked the NOK about the deceased’s race, offering the 5 IBGE categories as possible answers. We combined Black and *Pardo* groups into 1 category described as Black because these 2 racial groups experience more racism and adverse health outcomes compared with White people.^10,11^ According to Brazilian scholars and activists, using the dichotomous Black-White classification contributes to reducing racism and favors promoting equality.^10^ Also, the dichotomous classification of Black and White races facilitates the comparison with studies conducted in the United States.

Clinical assessment was performed using a semistructured questionnaire about previous medical diagnoses of hypertension, diabetes, coronary artery disease, heart failure, and stroke as well as lifestyle habits, such as smoking and alcohol use. Weight and height were measured before autopsy using an electronic scale and a stadiometer with the deceased in the supine position and without clothes.

Cognitive and functional abilities 3 months before death were evaluated using the informant part of the Clinical Dementia Rating (CDR),^12^ which evaluates 6 domains: memory, orientation, judgment and problem-solving, community affairs, home and hobbies, and personal care. Participants were classified into 5 categories: 0 (no dementia), 0.5 (questionable dementia), 1 (mild dementia), 2 (moderate dementia), and 3 (severe dementia). The outcome of cognitive impairment was defined as a CDR greater than 0.

Apolipoprotein E (APOE) genotypes were determined by allele-specific amplification (single-nucleotide variants rs429358 and rs7412) using real-time polymerase chain reaction assays.^13^ APOE genotypes were categorized into 2 groups: without an APOE4 allele ε4 (APOE4) and with at least 1 APOE4.

### Neuropathology

Selected areas from the right brain hemisphere were frozen at −80 °C, while the left hemisphere was fixed in 4% paraformaldehyde. Samples from the following areas were embedded in paraffin for pathological staining and analysis: middle and inferior frontal gyrus, middle and superior temporal gyri, angular gyrus, superior frontal and anterior cingulate gyrus, visual cortex, anterior hippocampus, hippocampal formation at the level of the lateral geniculate body, amygdala, basal ganglia at the level of the anterior commissure, thalamus, midbrain, pons, medulla oblongata, and cerebellum. Slides with 5 μm–thickness from these samples were stained with hematoxylin and eosin. Immunohistochemistry for β-amyloid (4G8,1:10.000; BioLegend#800701), phosphorylated tau (AT8,1:400; Invitrogen MN1020), transactive response DNA-binding protein 43 kDa (TDP-43) (1:500,BioLegend#829901), and α-synuclein (1:500; Biolegend #829901-BL) were performed in selected sections.^8^

AD pathology was scored using the Braak and Braak staging for neurofibrillary tangles,^14^ and the Consortium to Establish a Registry for AD (CERAD) for neuritic plaques.^15^ The AD diagnosis required a Braak and Braak stage of III or greater and a CERAD score of moderate or frequent. Lewy body pathology (LBP) was classified using the Braak staging for Parkinson disease (PD) and grouped in 3 categories: 0 to II, III to IV, and V to VI.^16^ LBP diagnosis was ascertained when the Braak PD stage was III or greater.

Cerebrovascular lesions were evaluated macroscopically during the sampling of the fixed and fresh brain areas and microscopically in the sampled areas. Large and lacunar infarcts were registered by location, size, and number. The infarct variable was considered present when 1 large infarct (≥1 cm) or 3 lacunar infarcts (<1 cm) were detected in 3 or more cortical areas. Infarcts were also present when detected in at least 1 strategic area (thalamus, frontal-cingular cortex, basal forebrain, caudate, medial temporal area, or angular gyrus). Small vessel disease (SVD), which included arteriolosclerosis and lipohyalinosis, was scored regarding location, extension, and severity of vessel changes. SVD was considered present when moderate or severe vascular changes were present in at least 3 cortical regions. Cerebral amyloid angiopathy (CAA) was evaluated according to location (meningeal, gray matter, and/or white matter) and severity of capillary amyloid deposition. CAA was present when it was detected in at least 3 different cortical areas. Vascular dementia (VD) diagnosis was ascertained when any of the criteria for lacunar infarct, SVD, or CAA were present.^17^ We considered the diagnoses of mixed dementia in the presence of the combination of AD and VD or AD and LBD pathology in the same participant. Other dementia diagnoses include less frequent diseases, such as progressive supranuclear palsy, corticobasal degeneration, frontotemporal dementia, and limbic-predominant age-related TDP-43 encephalopathy (LATE).

Siderocalcinosis, which is a vascular mineralization with an encrustation of calcium and iron in the middle layer, was evaluated in the basal ganglia and classified as present or absent.^17^ Hippocampal sclerosis was considered present when severe pyramidal cell loss and gliosis in the CA1 and subiculum were observed. TDP-43 deposition was evaluated in the hippocampus, amygdala, and middle frontal gyrus.^18^ TDP-43 staining was incorporated recently in the BAS immunohistochemistry panel. Currently, 1197 participants have TDP-43 evaluation.

To create a neuropathologic comorbidity index (NCI), we first investigated the association between each neuropathology and cognitive impairment using a logistic regression model adjusted for age, sex, and education. Since each neuropathological lesion was associated with different odds of dementia, we assigned points to each neuropathological variable by dividing each coefficient by the lowest coefficient and rounding up or down to the nearest integer.^8^ The NCI was calculated by summing the points for each variable and ranged from 0 to 10.

### Statistical Analysis

We compared sociodemographic and clinical variables by race (Black and White participants) using the χ^2^ test for categorical variables and the unpaired *t* test for continuous variables. The associations between race and neuropathology were investigated using ordinal logistic regression models for ordinal outcomes (Braak and Braak staging for neurofibrillary tangles, CERAD for neuritic plaques, and Braak staging for LBP) and logistic regression models for binary outcomes (infarcts, SVD, CAA, TDP-43, hippocampal sclerosis, and siderocalcinosis). The ordinal proportional odds assumption was tested using the likelihood ratio and the Brant tests.^19^ Models were first adjusted for age and sex, and then further adjusted for education, which is related to socioeconomic level and cognitive reserve.^20^ Next, we adjusted the models for clinical variables (hypertension, diabetes, dyslipidemia, coronary artery disease, heart failure, stroke, smoking, alcohol use, and body mass index). The model adjusted for clinical variables should be interpreted with caution because clinical variables could be mediators in the association between race and neuropathology. Finally, the associations were adjusted for APOE4. In addition, we examined the associations between race and neuropathological diagnoses using multinomial logistic regression in participants with these diagnoses: AD, VD, LBP, AD and VD, AD and LBP, and other diagnoses.

Moreover, we examined the association between race and cognitive impairment using logistic regression models, which were adjusted for the variables described previously. To examine the independent association between race and cognitive impairment from neuropathology, we further adjusted the models for the presence of pathologic lesions. To investigate the hypothesis that race could be a modifier in the association between neuropathology and cognitive impairment, we added interaction terms between race and each neuropathology on logistic regression models that had cognitive impairment as the dependent variable and each neuropathology as the independent variable and were adjusted for age, sex, and education.

We investigated the association of the NCI with race and cognitive impairment using ordinal regression models adjusted for age, sex, and education. We also examined whether the association between cognitive impairment and the NCI was modified by race by adding an interaction term of race and the NCI to the previous linear model. The α level was set at 5%, and all tests were 2-tailed. We used Stata version 15.0 (StataCorp) for statistical analyses.

## Results

From the original sample of 1990 decedents, we excluded 41 Asian individuals and 134 individuals with missing data for study variables (eFigure 1 in Supplement 1). Participants who were included in the study were younger and had less dyslipidemia. The frequencies of racial groups were similar between included and excluded participants, except for Asian participants because of the exclusion criteria (eTable 1 in Supplement 1). We analyzed data from 1815 participants; 617 (34%) were Black and 1198 (66%) were White, and 637 (35%) had cognitive impairment. White participants were older, had more years of education, and had a lower frequency of hypertension, stroke, alcohol consumption, and smoking than Black participants. Having at least 1 APOE4 was more common among Black than White participants (Table 1).

**Table 1.** Characteristics of the Sample by Race (n = 1815)

Characteristics	Participants, No. (%)			*P* value
	Total (N = 1815)	Black (n = 617)	White (n = 1198)	
Age, mean (SD), y^a^	74.0 (12.5)	72.1 (13.1)	75.0 (12.1)	<.001
Sex^b^				
Men	912 (50.2)	299 (48.5)	613 (51.2)	.27
Women	903 (49.8)	318 (51.5)	585 (48.8)	
Education, mean (SD), y^a^	4.7 (4.1)	4.1 (3.8)	5.0 (4.2)	<.001
Hypertension^b^	1172 (64.6)	423 (68.6)	749 (62.6)	.01
Diabetes^b^	522 (28.8)	168 (27.2)	354 (29.6)	.30
Dyslipidemia^b^	231 (12.7)	72 (11.7)	159 (13.3)	.33
Coronary artery disease^b^	360 (19.9)	121 (19.6)	239 (20.0)	.86
Heart failure^b^	307 (16.9)	110 (17.8)	197 (16.5)	.46
Stroke^b^	260 (14.3)	107 (17.3)	153 (12.8)	.009
Smoking^b^				
Never	903 (49.8)	281 (45.5)	622 (52.0)	.02
Current	390 (21.5)	152 (24.6)	238 (19.9)	
Former	521 (28.7)	184 (29.9)	337 (28.1)	
Alcohol use^b^				
Never or sometimes	1327 (73.2)	396 (64.2)	931 (77.8)	<.001
Current heavy use	133 (7.3)	67 (10.9)	66 (5.5)	
Former heavy use	354 (19.5)	154 (24.9)	200 (16.7)	
Body mass index, mean (SD)^a^^,^^c^	22.9 (5.2)	22.6 (5.4)	23.1 (5.0)	.07
CDR^b^				
0	1178 (64.9)	401 (65.0)	777 (64.9)	.22
0.5	188 (10.4)	70 (11.4)	118 (9.8)	
1	104 (5.7)	36 (5.8)	68 (5.7)	
2	97 (5.3)	39 (6.3)	58 (4.8)	
3	248 (13.7)	71 (11.5)	177 (14.8)	
Cognitive impairment (CDR >0)^b^	637 (35.1)	216 (35.0)	35.1 (421)	.96
Any apolipoprotein E allele ε4^b^	289 (28.8)	120 (36.4)	169 (25.0)	<.001

Abbreviation: CDR, Clinical Dementia Rating scale.

^a^ Unpaired *t* test.

^b^ χ^2^ test.

^c^ Body mass index calculated as weight in kilograms divided by height in meters squared.

**Figure 1.**
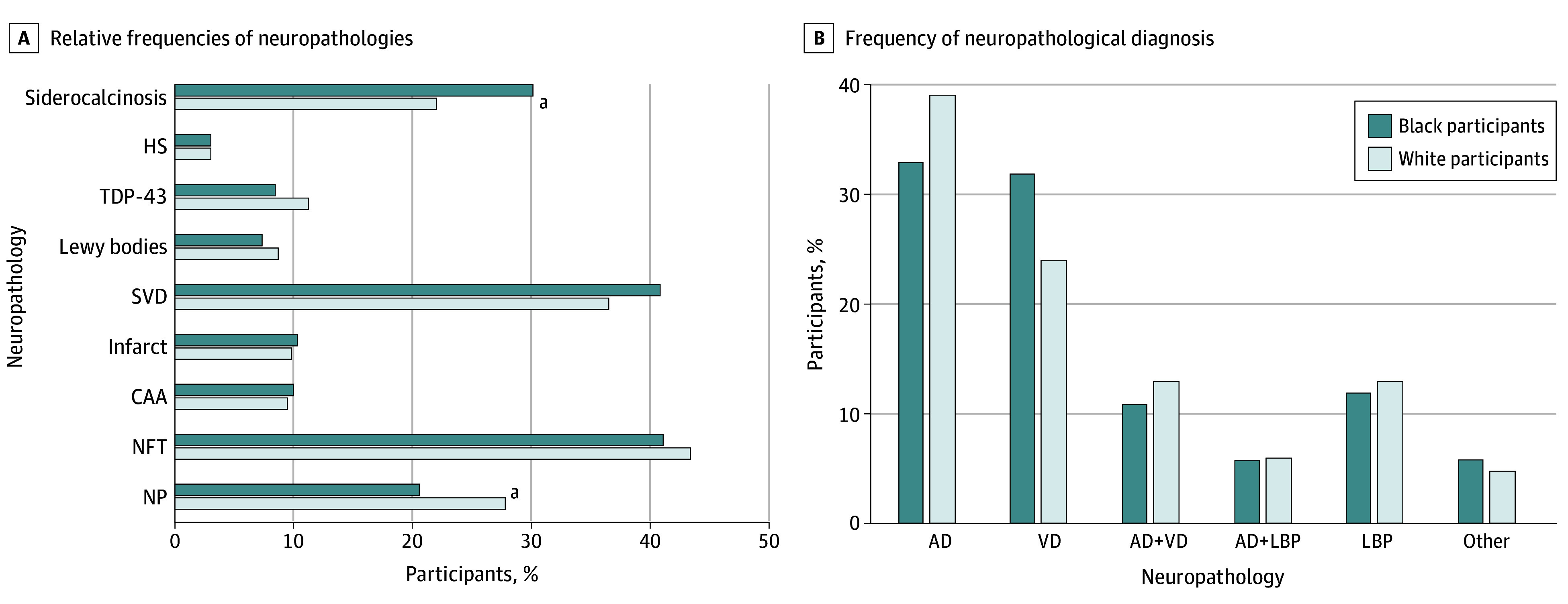
Frequencies of Neuropathologies and Neuropathological Diagnoses in Black and White Participants A total of 747 participants had a neuropathological diagnosis. AD indicates Alzheimer disease; CAA, cerebral amyloid angiopathy; HS, hippocampal sclerosis; LBP, Lewy body pathology; NFT, neurofibrillary tangles (Braak and Braak staging ≥III); NP, neuritic plaques (Consortium to Establish a Registry for Alzheimer Disease grade ≥B); SVD, small vessel disease; TDP-43, TAR DNA-binding protein 43; and VD, vascular dementia. ^a^*P* < .05.

When we compared the frequency of neuropathologic lesions by race in univariate analyses, we observed that neuritic plaques were less frequent in Black participants than White participants (126 [21%] vs 331 [28%]; *P* < .001), while siderocalcinosis was more frequent in Black than White participants (176 [30%] vs 256 [22%]; *P* < .001) (Figure 1A). In adjusted analyses for sociodemographic, clinical variables, and APOE4, Black participants had a lower frequency of neuritic plaques than White ones (odds ratio [OR], 0.61; 95% CI, 0.44-0.83; *P* = .002), and 74% higher odds of having SVD than White participants in adjusted analyses (OR, 1.74; 95% CI, 1.29-2.35; *P* < .001) (Table 2). Siderocalcinosis was also more frequent in Black than in White participants (OR, 1.70; 95% CI, 1.23-2.34, *P* = .001) (Table 2). Neurofibrillary tangles, LBP, TDP-43, hippocampal sclerosis, and other cerebrovascular lesions were not associated with race.

**Table 2.** Association Between Neuropathology and Race Among 1815 Participants^a^

Neuropathology	Unadjusted		Model 1		Model 2		Model 3		Model 4	
	Odds ratio (95% CI)	*P* value	Odds ratio (95% CI)	*P* value	Odds ratio (95% CI)	*P* value	Odds ratio (95% CI)	*P* value	Odds ratio (95% CI)	*P* value
Neuritic plaques^b^	0.65 (0.53-0.79)	<.001	0.76 (0.61-0.94)	.01	0.78 (0.63-0.97)	.02	0.78 (0.62-0.97)	.03	0.61 (0.44-0.83)	.002
Neurofibrillary tangles^b^	0.86 (0.72-1.02)	.08	1.06 (0.89-1.27)	.49	1.08 (0.90-1.29)	.39	1.05 (0.88-1.26)	.60	0.99 (0.78-1.27)	.96
Cerebral amyloid angiopathy^c^	1.06 (0.77-1.48)	.71	1.23 (0.88-1.72)	.24	1.26 (0.90-1.77)	.18	1.27 (0.89-1.80)	.19	1.22 (0.77-1.94)	.39
Infarct^b^	1.05 (0.76-1.45)	.77	1.15 (0.83-1.59)	.41	1.07 (0.77-1.48)	.70	0.98 (0.70-1.38)	.92	0.88 (0.54-1.41)	.58
Small vessel disease^b^	1.20 (0.98-1.47)	.08	1.33 (1.08-1.65)	.007	1.38 (1.11-1.70)	.003	1.28 (1.02-1.59)	.03	1.74 (1.29-2.35)	<.001
Lewy bodies^b^^,^^d^	0.84 (0.58-1.21)	.34	0.96 (0.66-1.40)	.83	1.00 (0.68-1.46)	>.99	1.09 (0.74-1.60)	.68	1.05 (0.63-1.76)	.85
TDP-43^b^^,^^e^	0.73 (0.49-1.09)	.12	0.83 (0.55-1.26)	.39	0.81 (0.54-1.24)	.34	0.85 (0.55-1.30)	.46	0.60 (0.32-1.12)	.11
Hippocampal sclerosis^b^	1.00 (0.57-1.76)	>.99	1.14 (0.64-2.01)	.66	1.11 (0.62-1.97)	.72	1.00 (0.55-1.80)	.99	0.98 (0.40-2.42)	.96
Siderocalcinosis^b^	1.52 (1.21-1.90)	<.001	1.65 (1.31-2.08)	<.001	1.68 (1.33-2.12)	<.001	1.58 (1.25-2.01)	<.001	1.70 (1.23-2.34)	.001

Abbreviation: TDP-43, TAR DNA-binding protein 43.

^a^ The reference group was White participants (n = 1198). Model 1 was adjusted for age and sex; model 2, adjusted for age, sex, and education; model 3, adjusted for age, sex, education, hypertension, diabetes, dyslipidemia, coronary artery disease, heart failure, stroke, smoking, alcohol use, and body mass index; model 4, adjusted for age, sex, education, hypertension, diabetes, dyslipidemia, coronary artery disease, heart failure, stroke, smoking, alcohol use, body mass index, and apolipoprotein E allele ε4. Model 4 included 1197 participants.

^b^ Ordinal logistic regression.

^c^ Binary logistic regression.

^d^ Lewy bodies were classified according to Braak staging for Parkinson disease and categorized as 0 to II, III to IV, and V to VI.

^e^ A total of 1197 participants were included in this analysis.

Among 747 participants who had neuropathological diagnoses, AD was the most frequent diagnosis (275 participants [37%]), followed by VD (197 participants [26%]) and mixed dementia of AD and VD (93 participants [12%]). When we compared the frequencies of neuropathological diagnoses by race, AD was more common among White participants than Black participants (198 [39%] vs 77 [33%]), while VD was more frequent among Black than White individuals (76 [32%] vs 121 [24%]) (Figure 1B). In multinomial logistic regression having neuropathological diagnoses as the outcome and race as the exposure variable, Black participants had a 61% higher risk of having VD than AD compared with White participants (relative risk, 1.61; 95% CI. 1.09-2.38; *P* = .02) (eTable 2 in Supplement 1).

**Figure 2.**
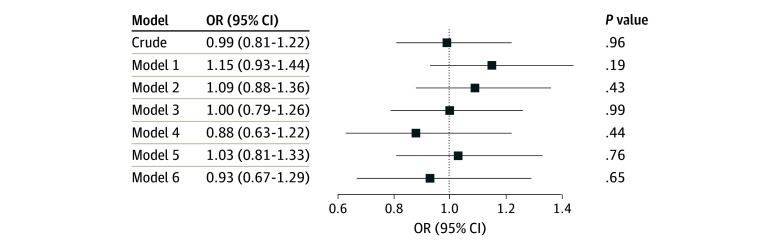
Association Between Cognitive Impairment and Race Evaluated Using Logistic Regression Models Adjusted for Age and Sex Model 1 was adjusted for age and sex; model 2, age, sex, and education; model 3, age, sex, education, and clinical variables (hypertension, diabetes, dyslipidemia, coronary artery disease, heart failure, stroke, smoking, alcohol use, and body mass index); model 4, age, sex, education, clinical variables, and apolipoprotein E allele ε4; model 5, age, sex, education, and neuropathology (neurofibrillary tangles, neuritic plaques, Lewy body pathology, cerebral amyloid angiopathy, small vessel disease, and siderocalcinosis); and model 6, age, sex, education, and neuropathology plus TAR DNA-binding protein 43. OR indicates odds ratio.

**Figure 3.**
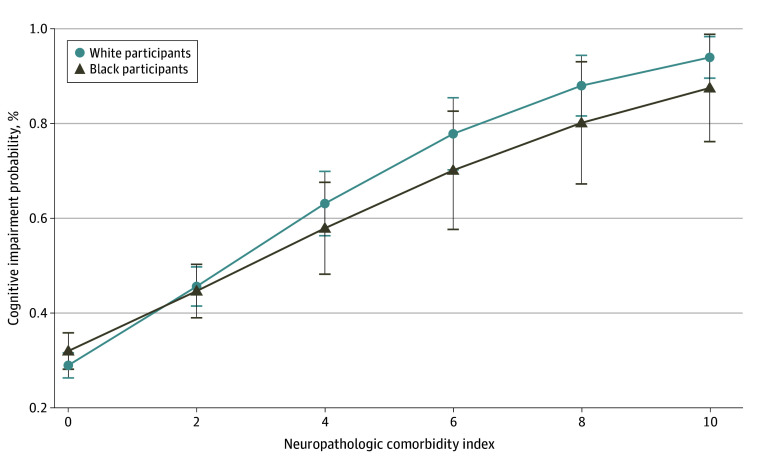
Association Between the Neuropathological Comorbidity Index and Cognitive Impairment by Race Logistic regression model adjusted for age, sex, and race and included an interaction term between race and the neuropathological comorbidity index (*P* = .16).

Race was not associated with cognitive impairment (Figure 2; eTable 3 in Supplement 1). Moreover, race was not an effect modifier in the association between neuropathologies and cognitive impairment given that the interactions between race and each pathology were not significant (eFigure 2 in Supplement 1). As expected, a higher NCI was associated with cognitive impairment (OR, 1.53; 95% CI, 1.44-1.63; *P* < .001). However, Black race was not associated with NCI scores in a model adjusted for age, sex, and education (OR, 1.14; 95% CI, 0.94-1.39; *P* = .18). Besides, the association between NCI and cognitive impairment was not modified by race (Figure 3).

## Discussion

In a large and diverse sample with an extensive neuropathological evaluation, we found race differences in the frequency of neuropathologies. SVD and siderocalcinosis were more frequent in Black participants, while neuritic plaques were more frequent in White participants. Likewise, AD diagnosis was more frequent in White participants, while VD was more common among Black participants. Race was not associated with cognitive impairment or NCI scores, and it was not an effect modifier in the association between neuropathology and cognition.

Neuropathological studies in diverse samples can assist the development of targeted and culturally sensitive diagnostic and treatment approaches for dementia and contribute to reducing health disparities. However, previous studies included mostly White individuals,^5,6,21,22^ which limits the generalizability of findings to diverse populations. Moreover, the majority of studies used convenience samples with patients with advanced dementia,^5,6,21,23^ which introduces selection bias given that brain donations in these settings depend on volunteerism, health access, trust in the health system, and misconceptions about AD symptoms being part of normal aging.^4^ Our population-based sample included individuals with normal cognition to advanced dementia and a large number of Black participants, which is important to fostering knowledge about neuropathology in diverse populations because when population-based samples were used, few racial differences were found.^24,25^ Moreover, we only studied individuals dwelling in a single city, minimizing environmental confounders. However, it is important to note that previous studies were conducted mostly in the United States, where the concept of race differs somewhat from the one adopted in Brazil. Although race encompasses ancestry and social components in both settings, differences in race concepts should be considered when comparing results. While race was mainly defined based on skin color (phenotypic component) and a category for mixed race was present in most Brazilian censuses, race was based on ancestry (genetic component) and multiracial categories were introduced only recently in the US census.^10^

Neuritic plaques were less frequent in Black participants compared with White ones, while SVD and siderocalcinosis were more frequent. To our knowledge, the lower frequency of neuritic plaques is a finding unique to our sample compared with other studies, which mostly did not find differences between Black and White participants.^21,26,27,28^ Conversely, 1 study with 2500 White and 100 African American participants found a higher burden of neurofibrillary tangle and neuritic plaque in African American decedents.^5^ Our finding of lower frequency of neuritic plaques could be related to differences in sample sizes or environmental and genetic risks for β-amyloid accumulation in diverse samples. Other studies had small samples of Black individuals (10-100 participants). The exact number of Black participants in the neuropathological study of Mehta et al,^21^ which had an overall sample size of 3017, was not reported. But if the proportion of Black participants from the main study, which included 30 916 patients, was maintained in the neuropathological study, 348 Black participants would have undergone neuropathological evaluation. Regarding environmental and genetic risks for β-amyloid accumulation, we have shown previously that African ancestry determined by genetic analyses was associated with lower odds of neuritic plaque accumulation.^7,29^ Besides, some environmental protective factors that may impact β-amyloid deposition may be more frequent in Brazil than in other regions, such as diet.^30^

SVD and siderocalcinosis were more frequent among Black than White participants in our sample. Again, previous studies presented mixed results regarding cerebrovascular lesions. While several studies did not find racial differences in frequencies of cerebrovascular lesions,^21,23,27,28,31^ others had found certain lesions to be more frequent in Black compared with White individuals.^5,32,33^ Similar to the study by Filshtein et al,^33^ we found that SVD was more frequent among Black participants. SVD frequency was also higher in Black than in White individuals in 2 previous studies,^5,33^ but this difference was not statistically significant probably due to the small sample of Black participants. We did not find racial differences in other cerebrovascular lesions, such as lacunar infarcts and CAA, which have been reported previously.^5,33^

Although siderocalcinosis has been related to cognitive impairment in our previous studies,^8,34^ siderocalcinosis was not reported by other studies, which makes impossible the comparison of our finding of siderocalcinosis being more frequent in Black participants. Siderocalcinosis is a vascular mineralization with an encrustation of calcium and iron in the elastic fibers of the media layer of the cerebral arteries, which is often regarded as a normal aspect of aging.^35,36^ Further studies on the racial differences of siderocalcinosis are needed. Similar to other studies,^24,25,31^ we did not find differences in the frequencies of LBP, CAA, and TPD-43.

AD was the most frequent neuropathological diagnosis in both racial groups, followed by VD and mixed pathology of AD plus VD. The relative frequency of AD was lower in Black participants, while VD had a larger frequency—almost equaling the frequency of AD—in Black participants. Similar findings were reported by Barnes et al,^6^ who found that pure AD was more common in White individuals. de la Monte et al^32^ also found more AD diagnosis and less VD in White participants, while Filshtein et al^33^ found VD to be more frequent in Black participants.

Although the frequencies of some neuropathologies were more frequent in Black participants, we did not find differences in cognitive impairment frequency by race. Similar cognitive abilities by race were reported by other studies,^6,24^ while in another study African American participants had lower cognitive scores in the clinical visit before death.^5^ In 2 cross-sectional studies,^37,38^ Black individuals had greater cognitive impairment than White individuals, but socioeconomic factors may have influenced test performance and interpretation since most normative data were developed and validated in White populations. Nevertheless, longitudinal studies showed mixed findings for race differences on cognitive decline, with some studies showing no difference,^39,40^ and others showing faster or slower declines in Black compared with White individuals.^41,42^ Although this study is cross-sectional, we could not capture racial differences in the CDR scores, which measure cognitive and functional abilities instead of actual cognitive performance. Moreover, no effect modification of race on the association between pathology and cognition was found. The interaction of race and neuropathologies has not been investigated, probably due to the large sample sizes that are required for these analyses.

### Limitations

Although we presented novel data on race differences in a large neuropathological study, our study has limitations. We did not monitor participants prior to their death, but cognitive assessments were conducted through an informant, who had regular, at least weekly contact with the deceased. Moreover, we conducted a validation study^43^ in which we compared the cognitive evaluation with an informant as performed in this study to a complete evaluation performed by specialists in dementia with patients and their informants. This study demonstrated high accuracy in diagnosing dementia through postmortem evaluations. Another limitation is the missing data for TDP-43 (34% missingness) and APOE genotyping (45%). TDP-43 staining was not available and APOE genotype was not included when the collection was initiated in 2004. Although our study was population-based, selection bias may still be present due to factors related to study eligibility and selection to compulsory autopsy. First, age and dyslipidemia differed between included and excluded individuals. Second, although the sample had similar age and sex to the 2010 Brazilian census, race composition differed: 54% were reported as White in our study, while 45% were self-reported as White in the census.^44^ This difference in race proportions may be related to the fact that the race reported by an informant may not be the same as the self-reported race by the deceased. Finally, our sample is from Sao Paulo, the largest city in Brazil, and the associations found in this study may not hold in other settings, like rural areas. Further studies in diverse settings and countries are needed.

## Conclusions

In this cross-sectional autopsy study of older Brazilians, neuritic plaques were less frequent in Black participants compared with White participants, while SVD and siderocalcinosis were more frequent. Cognitive and functional abilities and NCI scores were similar between race groups, and race was not an effect modifier in the associations of cognition with neuropathologies. Further neuropathological studies in diverse samples are important to decrease race disparities in dementia burden.

## Notes

Supplement 1. **eFigure 1.** Flowchart of the Study Participants **eFigure 2.** Association Between Cognitive Impairment and Neuropathological Lesions by Race **eTable 1.** Comparison Between Included and Excluded Individuals From the Study **eTable 2.** Association Between Neuropathological Diagnoses and Race **eTable 3.** Association Between Cognitive Impairment and Race **eTable 4.** Association Between Cognitive Impairment and Neuropathological Lesions

Supplement 2.
Data Sharing Statement
