# Supplementary material for: Neuropathological Lesions and Cognitive Abilities in Black and White Older Adults in Brazil
Source: JAMA Netw Open. 2024 Jul 25;7(7):e2423377. doi: 10.1001/jamanetworkopen.2024.23377 (PMC11273230; doi:10.1001/jamanetworkopen.2024.23377)
Supplement: Supplement 1. — eFigure 1. Flowchart of the Study Participants eFigure 2. Association Between Cognitive Impairment and Neuropathological Lesions by Race eTable 1. Comparison Between Included and Excluded Individuals From the Study eTable 2. Association Between Neuropathological Diagnoses and Race eTable 3. Association Between Cognitive Impairment and Race eTable 4. Association Between Cognitive Impairment and Neuropathological Lesions [file jamanetwopen-e2423377-s001.pdf]

# Supplemental Online Content

Suemoto CK, Leite RP, Paes VR, et al. Neuropathological lesions and cognitive abilities in Black and White older adults. *JAMA Netw Open.* 2024;7(7):e2423377. doi:10.1001/jamanetworkopen.2024.23377

**eFigure 1.** Flowchart of the Study Participants

**eFigure 2.** Association Between Cognitive Impairment and Neuropathological Lesions by Race

**eTable 1.** Comparison Between Included and Excluded Individuals From the Study

**eTable 2.** Association Between Neuropathological Diagnoses and Race

**eTable 3.** Association Between Cognitive Impairment and Race

**eTable 4.** Association Between Cognitive Impairment and Neuropathological Lesions

This supplemental material has been provided by the authors to give readers additional information about their work.

© 2024 Suemoto CK et al. *JAMA Network Open*.

***Supplemental Figures***

**eFigure 1**. Flowchart of the Study Participants

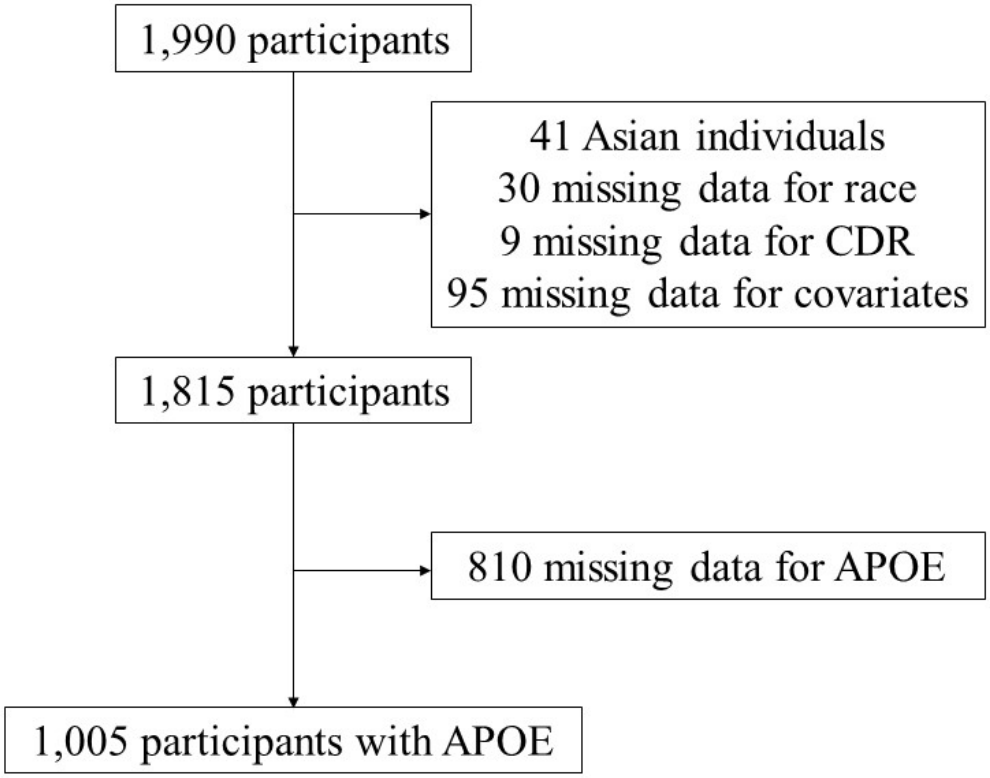


© 2024 Suemoto CK et al. *JAMA Network Open*.

**eFigure 2.** Association Between Cognitive Impairment and Neuropathological Lesions by Race

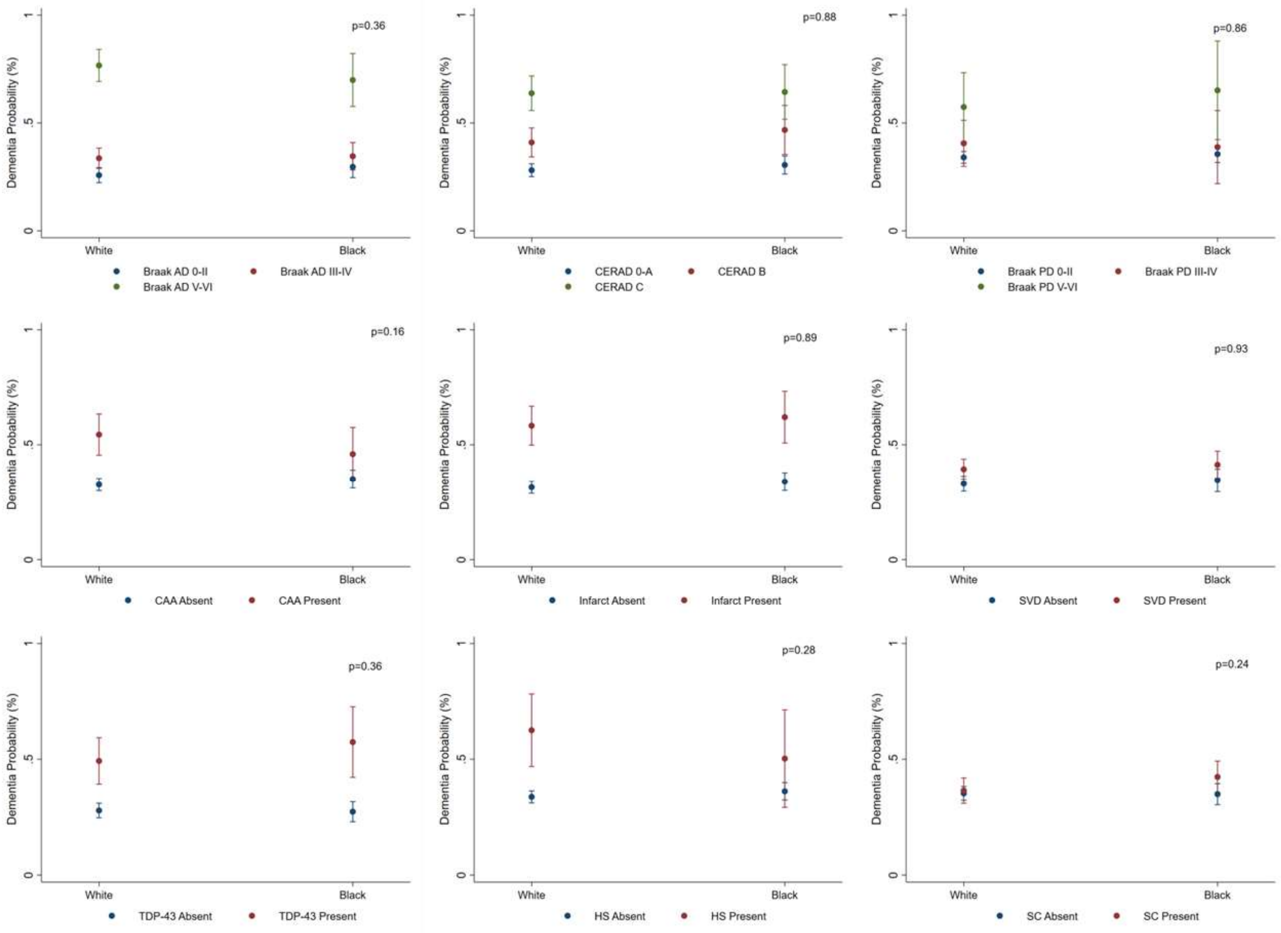


Linear regression models adjusted for age, sex, and education. P-values for the interaction terms between race and each neuropathology. Braak AD: Braak and Braak staging for neurofibrillary tangles; CERAD: Consortium to Establish a Registry for Alzheimer's Disease for neuritic plaques; Braak PD: Braak staging for Parkinson's disease; CAA: cerebral amyloid angiopathy; SVD: small vessel disease; TDP-43: TAR DNA-binding protein 43; HS: hippocampal sclerosis; SC: siderocalcinosis.

© 2024 Suemoto CK et al. *JAMA Network Open*.

eTable 1. Comparison Between Included and Excluded Individuals From the Study

| Variable | Included n=1,815 | Excluded n=175 | p |
|---|---|---|---|
| **Age (years), mean (SD)**[ǁ] | 74 (13) | 78 (13) | <0.001 |
| **Men, %**[├] | 50% | 50% | 0.95 |
| **Education (years), mean (SD)**[ǁ] | 5 (4) | - | - |
| **Race, %**[├] | | | <0.001 |
| White | 66 | 47 | |
| Black | 12 | 11 | |
| Pardo | 22 | 19 | |
| Asian | 0 | 22 | |
| **Race without Asian, %**[├] | | | 0.45 |
| White | 66 | 61 | |
| Black | 12 | 15 | |
| Pardo | 22 | 24 | |
| **Hypertension, %**[├] | 65 | 70 | 0.15 |
| **Diabetes, %**[├] | 29 | 32 | 0.32 |
| **Dyslipidemia, %**[├] | 13 | 27 | <0.001 |
| **Coronary artery disease, %**[├] | 20 | 17 | 0.39 |
| **Heart failure, %**[├] | 17 | 14 | 0.30 |
| **Stroke, %**[├] | 14 | 16 | 0.64 |
| **Smoking, %**[├] | | | 0.35 |
| Never | 50 | 55 | |
| Current | 21 | 18 | |
| Former | 29 | 27 | |
| **Alcohol use, %**[├] | | | 0.93 |
| Never/Sometimes | 73.1 | 72.4 | |
| Current heavy use | 7.3 | 6.9 | |
| Former heavy use | 19.6 | 20.7 | |
| **Body mass index, mean (SD)**[ǁ] | 23 (5) | 22 (5) | 0.23 |
| **Clinical Dementia Rating (CDR), %**[├] | | | 0.35 |
| **0** | 65% | 59% | |
| **0.5** | 10% | 11% | |
| **1** | 6% | 8% | |
| **2** | 5% | 4% | |
| **3** | 14 | 18 | |

SD: standard deviation

[ǁ]unpaired t-test; [├]chi-square test

© 2024 Suemoto CK et al. *JAMA Network Open*.

eTable 2. Association Between Neuropathological Diagnoses and Race* (n=747)

| Diagnoses | Relative Risk | 95% CI | p |
|---|---|---|---|
| **Alzheimer's disease** | Reference | | |
| **Vascular dementia** | 1.61 | 1.09-2.38 | 0.02 |
| **Lewy body pathology** | 1.12 | 0.67-1.89 | 0.66 |
| **Alzheimer's disease + vascular dementia** | 0.94 | 0.56-1.60 | 0.83 |
| **Alzheimer's disease + Lewy body pathology** | 1.17 | 0.60-2.27 | 0.65 |
| **Other** | 1.29 | 064-2.57 | 0.48 |

Black participants compared to White participants

© 2024 Suemoto CK et al. *JAMA Network Open*.

eTable 3. Association Between Cognitive Impairment and Race (n=1,815)

| | Odds Ratio | 95% CI | p |
|---|---|---|---|
| **Crude** | 0.99 | 0.81-1.22 | 0.96 |
| **Model 1** | 1.15 | 0.93-1.44 | 0.19 |
| **Model 2** | 1.09 | 0.88-1.36 | 0.43 |
| **Model 3** | 1.00 | 0.79-1.26 | 0.99 |
| **Model 4** | 0.88 | 0.63-1.22 | 0.44 |
| **Model 5** | 1.03 | 0.81-1.33 | 0.76 |
| **Model 6** | 0.93 | 0.67-1.29 | 0.65 |

Model 1: Logistic regression model adjusted for age and sex
Model 2: Logistic regression model adjusted for age, sex, and education
Model 3: Logistic regression model adjusted for age, sex, education, hypertension, diabetes, dyslipidemia, coronary artery disease, heart failure, stroke, smoking, alcohol use, and body mass index
Model 4: Logistic regression model adjusted for age, sex, education, hypertension, diabetes, dyslipidemia, coronary artery disease, heart failure, stroke, smoking, alcohol use, body mass index, and apolipoprotein E allele 4 (n=1,005)
Model 5: Logistic regression model adjusted for age, sex, education, Braak & Braak staging for neurofibrillary tangles, Consortium to Establish a Registry for Alzheimer's Disease (CERAD) score for neuritic plaques, Braak & Braak staging for Lewy pathology, infarcts, small vessel disease, amyloid cerebral angiopathy, siderocalcinosis, hippocampal sclerosis
Model 6: Logistic regression model adjusted for age, sex, education, Braak & Braak staging for neurofibrillary tangles, Consortium to Establish a Registry for Alzheimer's Disease (CERAD) score for neuritic plaques, Braak & Braak staging for Lewy pathology, infarcts, small vessel disease, amyloid cerebral angiopathy, siderocalcinosis, hippocampal sclerosis, and TDP-43 (n=1,197).

© 2024 Suemoto CK et al. *JAMA Network Open*.

eTable 4. Association Between Cognitive Impairment and Neuropathological Lesions (n=1,815)

| Neuropathology | OR (95% CI) | p | Points |
|---|---|---|---|
| **CERAD** | | 0.28 | |
| 0-A | 1 (reference) | | 0 |
| B | 1.23 (0.87-1.75) | | 0 |
| C | 1.42 (0.86-2.33) | | 0 |
| **Braak NFT** | | <0.001 | |
| 0-II | 1 (reference) | | 0 |
| III-IV | 1.29 (0.97-1.71) | | 1 |
| V-VI | 6.06 (3.55-10.36) | | 5 |
| **Cerebral amyloid angiopathy** | 1.58 (1.05-2.39) | 0.03 | 1 |
| **Infarct** | 3.60 (2.51-5.16) | <0.001 | 3 |
| **Small vessel disease** | 1.07 (0.83-1.37) | 0.44 | 0 |
| **Braak LB** | | 0.002 | |
| 0-II | 1 (reference) | | 0 |
| III-IV | 1.20 (0.74-1.96) | | 1 |
| V-VI | 3.13 (1.63-6.01) | | 3 |
| **Hippocampal sclerosis** | 2.28 (1.12-4.64) | 0.02 | 2 |
| **Siderocalcinosis** | 1.08 (0.83-1.41) | 0.57 | 0 |

OR: odds ratio

Logistic regression model adjusted for age, sex, and education

© 2024 Suemoto CK et al. *JAMA Network Open*.
