# Supplementary material for: Neuropathological Lesions and Cognitive Abilities in Black and White Older Adults in Brazil
Source: JAMA Netw Open. 2024 Jul 25;7(7):e2423377. doi: 10.1001/jamanetworkopen.2024.23377 (PMC11273230; doi:10.1001/jamanetworkopen.2024.23377)
Supplement: Supplement 2. — Data Sharing Statement [file jamanetwopen-e2423377-s002.pdf]

Data Sharing Statement

Suemoto. Neuropathological Lesions and Cognitive Abilities in Black and White Older Adults. *JAMA Netw Open*. Published July 25, 2024. doi:10.1001/jamanetworkopen.2024.23377

Data
Data available: Yes
Data types: Deidentified participant data, Data dictionary
How to access data: Deidentified data and data dictionary will be available upon request to the principal investigator.
When available: With publication

Supporting Documents
Document types: None

Additional Information
Who can access the data: Researchers whose proposed use of the data has been approved
Types of analyses: Data will be available for a specific purpose
Mechanisms of data availability: Data will be available with investigator support, after approval of a proposal, and with s signed data access agreement.
